# Rgg-Associated SHP Signaling Peptides Mediate Cross-Talk in Streptococci

**DOI:** 10.1371/journal.pone.0066042

**Published:** 2013-06-11

**Authors:** Betty Fleuchot, Alain Guillot, Christine Mézange, Colette Besset, Emilie Chambellon, Véronique Monnet, Rozenn Gardan

**Affiliations:** 1 INRA, UMR1319 MICALIS, Jouy en Josas, France; 2 AgroParistech, UMR MICALIS, Jouy en Josas, France; Ben-Gurion University of the Negev, Israel

## Abstract

We described a quorum-sensing mechanism in the streptococci genus involving a short hydrophobic peptide (SHP), which acts as a pheromone, and a transcriptional regulator belonging to the Rgg family. The *shp/rgg* genes, found in nearly all streptococcal genomes and in several copies in some, have been classified into three groups. We used a genetic approach to evaluate the functionality of the SHP/Rgg quorum-sensing mechanism, encoded by three selected *shp/rgg* loci, in pathogenic and non-pathogenic streptococci. We characterized the mature form of each SHP pheromone by mass-spectrometry. We produced synthetic peptides corresponding to these mature forms, and used them to study functional complementation and cross-talk between these different SHP/Rgg systems. We demonstrate that a SHP pheromone of one system can influence the activity of a different system. Interestingly, this does not seem to be dependent on the SHP/Rgg group and cross-talk between pathogenic and non-pathogenic streptococci is observed.

## Introduction

Quorum-sensing (QS) is a cell-cell communication mechanism that allows bacteria to control gene expression in a co-ordinated manner at a population scale. It involves the detection of an autoinducer signal that is synthesized, and actively or passively secreted; it is detected, or triggers a response, when its extracellular concentration reaches a threshold or quorum. This sensing leads cells to modulate expression of the gene targets of the mechanism. QS controls various important functions including for example virulence in *Pseudomonas aeruginosa* [1] and *Staphylococcus aureus* [2], biofilm development in *Pseudomonas putida* [3] and *S. aureus* [2] and sporulation in bacilli [4]. The autoinducers of Gram-negative bacteria mainly belong to the family of acyl-homoserine lactones [5] whereas in those of Gram-positive bacteria are peptides [6], [7].

Peptide autoinducers are either detected indirectly from the extracellular medium or directly in the intracellular medium. In the first case, the peptides are sensed by the histidine kinase of a two component system. This leads to the modification of the phosphorylation status of the histidine kinase and then of its cognate response regulator which modulates the expression of its target genes. This mechanism controls many functions, including triggering competence for natural transformation in *Streptococcus pneumoniae* and *Bacillus subtilis* [8]. In the second case, the peptides are imported into the cell by an oligopeptide transporter and once inside, interact with a transcriptional regulator or a Rap protein; conjugation in *Enterococcus faecalis* and virulence in *Bacillus cereus* [9] are controlled by this type of mechanism.

Many of these QS mechanisms have been deciphered in detail by more than 40 years of studies, such that we now understand quite well how a cell communicates with its siblings. A QS issue that has emerged more recently is the communication between different strains of the same species and even between species. Work on this subject has led to the definition of pherotypes or specificity groups, and amino acid sequence polymorphism has been documented for the signaling peptides and their receptors. All bacteria that belong to one pherotype can sense the peptides synthesized by members of the same pherotype but not the peptides synthesized by members of the others. Pherotypes have been defined for different mechanisms: Agr in *S. aureus* [10], [11], ComCDE in *S. pneumoniae* [12], ComQXPA in *B. subtilis* [13], [14] and PapR/PlcR in *B. cereus* [15], [16]. These studies are of significance for at least two reasons: i) a better knowledge of the interaction between the signaling peptides and their receptors may allow intervention, based on synthetic peptides, and this would be of particular value for the regulation of virulence factors as demonstrated in *S. aureus* [17]; ii) deciphering this diversity and evolution may help understand ecological adaptation by bacteria [18].

We recently discovered a QS mechanism that relies on a transcriptional regulator of the Rgg family and a small hydrophobic peptide (SHP) detected in the intracellular medium [19], [20]. We studied the *shp*/*ster_1358* (*rgg1358*) locus of *Streptococcus thermophilus* LMD-9, where the two genes are transcribed divergently, and showed that SHP1358 is secreted, matured and imported back into the cell by the oligopeptide transporter Ami. Then, the mature form of SHP1358 interacts with Rgg1358 enabling Rgg1358 to control, positively, the expression of two targets, *shp1358* and *ster_1357* [21]. The *ster_1357* gene encodes a secreted cyclic peptide of unknown function. A similar mechanism has been suggested for the *shp/stu0182* (*rgg0182*) locus of *S. thermophilus* strain LMG18311 [22] and has been confirmed for two SHP/Rgg systems in *Streptococcus pyogenes*: one is an activator and the other a repressor involved in biofilm development [23], [24]. The phylogenetic tree of Rgg-like proteins indicates that Rgg are widespread in Gram-positive bacteria but that *shp*-associated *rgg* genes are only found in the streptococci genus. This genus contains various species, including commensal bacteria of the human microbiome, the GRAS (generally recognized as safe) bacterium, *S. thermophilus*, used for the manufacture of dairy products, but also human pathogenic bacteria such as *S. pneumoniae*, *S. agalactiae*, *S. pyogenes* and *S. mutans* [25]. We found 68 *shp/rgg* copies, 28 of which encode a unique amino acid sequence, although the sequences of all these SHP pheromones are generally similar. Nearly all streptococci genomes contain one copy, but some streptococci have multiple copies, for example *S. thermophilus* strain LMD-9 has six. This phylogenetic study of Rgg amino acid sequences led to their classification into three groups. In groups I and II, the SHPs have a conserved glutamate and aspartate, respectively, and the *shp* and *rgg* genes are transcribed divergently. In group III, the *shp* genes are located downstream from the *rgg* genes, in a convergent orientation and the SHPs have a glutamate or an aspartate residue [21].

Different streptococci species can meet in raw milk [26], the human oral microbiome [27], [28] and gastrointestinal tract [29]. We therefore investigated whether there is interspecies cross-talk via SHP peptides. We first studied the functionality of the SHP/Rgg cell-cell communication mechanism associated with three different *shp/rgg* loci, one from each of the three groups, in three distinct streptococci species. The mature form of each SHP was identified in the extracellular medium. We then used synthetic peptides to study the specificity of the interaction between the SHPs and the Rgg regulators of different groups and species. We demonstrate cross-talk between SHP/Rgg systems belonging to distinct groups and different species.

## Materials and Methods

### Bacterial Strains and Growth Conditions

The bacterial strains used in this study are listed in Table 1. *S. thermophilus, S. agalactiae* and *S. mutans* strains were grown at 30, 37 or 42°C in M17 medium (Difco) supplemented with 10 g l^–1^ lactose (M17lac) or in a chemically defined medium (CDM) without shaking, under atmospheric air and with a ratio of air space to liquid of approximately 90% [30]. *Escherichia coli* strains were grown at 30 or 37°C in Luria-Bertani (LB) broth with shaking. Agar (1.5%) was added to the media as appropriate. When required, antibiotics were added to the media at the following final concentrations: erythromycin, 200 µg ml^–1^ for *E. coli* and 5 µg ml^–1^ for *S. thermophilus*; and kanamycin, 1 mg ml^–1^ for *S. thermophilus*. The optical density at 600 nm of the cultures was measured with an Uvikon 931 spectrophotometer (Kontron).

**Table 1:** Bacterial strains used in this study.

Bacterial strain and genotype			Resistance^a^	Description^b^		Source or reference		
***Streptococcus thermophilus*** ** LMD-9 and derivates**								
	LMD-9	Wild-type					[38]	
	TIL1038	*blp*::P*_shp1299_-luxAB aphA3*	Km		pGICB004a::P*_shp1299_* → LMD-9		This study	
	TIL1042	*amiCDE*::*erm blp*::P*_shp1299_-luxAB aphA3*	Km/Erm		TIL1389 DNA → TIL1038		This study	
	TIL1047	*shp1299*::*erm* c	Erm		PCR fragment *shp1299::erm* → LMD-9		This study	
	TIL1048	Δ*ster_1299 blp*::P*_shp1299_-luxAB aphA3*	Km		pGICB004a::P*_shp1299_* → TIL1160		This study	
	TIL1052	*shp1299*::*erm blp*::P*_shp1299_-luxAB aphA3*	Km/Erm		pGICB004a::P*_shp1299_* → TIL1047		This study	
	TIL1160	Δ*ster_1299*			pG^+^host9::updown.*ster_1299* → LMD-9		This study	
	TIL1165	*blp*::P*_shp1358_-luxAB*					[21]	
	TIL1200	Δ*shp1358 blp*::P*_shp1358_-luxAB*					[21]	
	TIL1345	*blp*::*gbs1555 shp1555*-*luxAB aphA3*	Km		pGICB004a::*gbs1555 shp1555* → LMD-9		This study	
	TIL1380	*blp*::*shp1555*-*luxAB aphA3*	Km		pGICB004a::*shp1555* → LMD-9		This study	
	TIL1381	*amiCDE::erm blp*::*gbs1555 shp1555*-*luxAB aphA3*	Km/Erm		TIL1389 DNA → TIL1345		This study	
	TIL1382	*blp*::*gbs1555* P*_shp1555_*-*luxAB aphA3*	Km		pGICB004a::*gbs1555* P*_shp1555_* → LMD-9		This study	
	TIL1383	*blp*::SMU.1509 *shp1509*-*luxAB aphA3*	Km		pGICB004a::SMU.1509 *shp1509* → LMD-9		This study	
	TIL1384	*blp*::SMU.1509 P*_shp1509_*-*luxAB aphA3*	Km		pGICB004a::SMU.1509 P*_shp1509_* → LMD-9		This study	
	TIL1385	*amiCDE::erm blp*::SMU.1509 *shp1509*-*luxAB aphA3*	Km/Erm		TIL1389 DNA → TIL1383		This study	
	TIL1386	*blp*::*shp1509*-*luxAB aphA3*	Km		pGICB004a::*shp1509* → LMD-9		This study	
	TIL1389	*amiCDE::erm*	Erm		PCR fragment *amiCDE::erm* → LMD-9		This study	
***Streptococcus mutans***								
	UA159	Wild-type ATCC 700610					[39]	
***Streptococcus agalactiae***								
	NEM316	Wild-type					[40]	
***Escherichia coli***								
	TG1 *repA^+^*	TG1 derivative with *repA* gene integrated into the chromosome					[19]	

a Km and Erm indicate resistance to kanamycin and erythromycin, respectively.

b Arrows indicate construction by transformation with chromosomal DNA or plasmid.

c *shp1299* is annotated *ster_1298* in Genbank.

### DNA Manipulation and Sequencing

Restriction enzymes, T4 DNA ligase (New England Biolabs), and Phusion DNA polymerase (Finnzymes) were used according to the manufacturers’ instructions. Standard methods were used for DNA purification, restriction digestion, PCR, ligation and sequencing. The oligonucleotides, purchased from Eurogentec, are listed in Table S1. PCR amplifications were carried out in a GeneAmp PCR System 2720 (Applied Biosystems) and all amplified fragments were purified with a Wizard purification kit (Promega). Plasmids were extracted with QIAprep spin miniprep kits (Qiagen). The *E. coli* strain TG1 *repA^+^* was used as the host for cloning experiments. *S. thermophilus* was transformed using natural competent [31] or electrocompetent [19] cells. The plasmids used are listed in Table 2.

**Table 2:** Plasmids used in this study.

Plasmid	Descriptiona,b	Source or reference
pG^+^host9	Erm, Ts plasmid	[41]
pG^+^host9::updown.*ster_1299*	Erm, pG^+^host9 derivative, for *ster_1299* gene remplacement by double cross-over integration	This study
pGICB004	Erm, Ts plasmid allowing the integration of transcriptional fusions to the *luxAB* reportergenes at the *blp* locus in *S. thermophilus*	[21]
pGICB004a	Erm, pGICB004 derivative containing the *aphA3* gene, conferring kanamycin resistance,upstream from the *luxAB* genes	This study
pGICB004a::P*_shp1299_*	Erm, Km, pGICB004a derivative used to introduce a P*_shp1299_*-*luxAB* transcriptional fusioninto *S. thermophilus* LMD-9	This study
pGICB004a::*gbs1555 shp1555*	Erm, Km, pGICB004a derivative used to introduce a *gbs1555 shp1555-luxAB* transcriptionalfusion into *S. thermophilus* LMD-9	This study
pGICB004a::*gbs1555* P*_shp1555_*	Erm, Km, pGICB004a derivative used to introduce a *gbs1555* P*_shp1555_*-*luxAB* transcriptionalfusion into *S. thermophilus* LMD-9	This study
pGICB004a::*shp1555*	Erm, Km, pGICB004a derivative used to introduce a *shp1555-luxAB* transcriptionalfusion into *S. thermophilus* LMD-9	This study
pGICB004a::*shp1509*	Erm, Km, pGICB004a derivative used to introduce a *shp1509-luxAB* transcriptionalfusion into *S. thermophilus* LMD-9	This study
pGICB004a::SMU.1509 *shp1509*	Erm, Km, pGICB004a derivative used to introduce a SMU.1509 *shp1509-luxAB* transcriptionalfusion into *S. thermophilus* LMD-9	This study
pGICB004a::SMU.1509 P*_shp1509_*	Erm, Km, pGICB004a derivative used to introduce a SMU.1509 P*_shp1509_*-*luxAB* transcriptionalfusion into *S. thermophilus* LMD-9	This study

a Ts indicates that the plasmid encodes a thermosensitive RepA protein.

b Km and Erm indicate resistance to kanamycin and erythromycin, respectively.

### Construction of Mutant Strains

The overlapping PCR method was used to delete the *shp1299* and the *amiCDE* genes as follows. Briefly, the erythromycin (erm) cassette was amplified by PCR with oligonucleotides Erm-F and Erm-R and pG^+^host9 as the template and fused by PCR to fragments located upstream and downstream from the *shp1299* gene and to fragments located upstream from the *amiC* gene and downstream from the *amiE* gene. Upstream and downstream fragments of both *shp1299* and *ami* genes were amplified with oligonucleotides shp1299_up-F/shp1299_up-R and shp1299_down-F/shp1299_down-R and amiCDE_up-F/amiCDE_up-R, amiCDE_down-F/amiCDE_down-R. The resulting fused PCR fragments were used to transform natural competent cells of strain LMD-9 leading to the construction of strains TIL1047 (*shp1299::erm*) and TIL1389 (*amiCDE::erm*). Strain TIL1160 (Δ*ster_1299*) was constructed by deleting an internal fragment of the gene by a double crossover event using pG^+^host9. Briefly, oligonucleotides ster_1299-SpeI with ster_1299-EcoRIA and ster_1299-EcoRIB with ster_1299-HindIII were used to amplify upstream and downstream fragments from the *ster_1299* gene; the resulting two fragments were double digested with the restriction enzymes *Spe*I+*Eco*RI and *Eco*RI+*Hind*III, respectively, and ligated between the *Spe*I and *Hind*III restriction sites of pG^+^host9. The resulting plasmid, pG^+^host9::updown.*ster_1299*, was used to transform electrocompetent cells of strain LMD-9. Integration and excision of the plasmid led to the deletion of the *ster_1299* gene.

### Constructions of Strains Containing *luxAB* Reporters

First, plasmid pGICB004a, a derivative of pGICB004, was constructed to facilitate integration of transcriptional fusions to the *luxAB* reporter genes into the *blp* locus in *S. thermophilus* LMD-9 by natural transformation and double crossover events. For this purpose, the *aphA3* cassette was amplified with oligonucleotides AphA3-F and AphA3-R using plasmid pKa as the template [32]. The resulting fragment was inserted at the *Sma*I restriction site, downstream from the *luxAB* genes in pGICB004. To study the expression of the *shp1299*, *shp1555* and *shp1509* genes in various genetic backgrounds, derivatives of pGICB004a were constructed and used to transform various strains of *S. thermophilus* as described below. The plasmid, pGICB004a::P*_shp1299_*, was constructed as follows. The *shp1299* promoter was amplified with oligonucleotides Pshp1299-EcoRI and Pshp1299-SpeI, double digested with the restriction enzymes *Spe*I and *Eco*RI and ligated between the same restriction sites of pGICB004a. *Sca*I-linearized pGICB004a::P*_shp1299_* was used to transform competent cells of strains LMD-9, TIL1047 and TIL1160 leading to strains TIL1038 (*blp*::P*_shp1299_-luxAB aphA3*), TIL1052 (*shp1299*::*erm blp*::P*_shp1299_-luxAB aphA3*) and TIL1048 (Δ*ster_1299 blp*::P*_shp1299_-luxAB aphA3*), respectively. The plasmids pGICB004a::*gbs1555 shp1555*, pGICB004a::*gbs1555* P*_shp1555_* and pGICB004a::*shp1555* were constructed similarly and in these cases, the PCR fragments ligated into each plasmid were amplified with oligonucleotides GBS-SpeI/GBS-EcoRI, GBS-SpeI/GBSshp-EcoRI and GBSrgg-SpeI/GBS-EcoRI, respectively. Natural transformation of strain LMD-9 with each linearized plasmid lead to construction of strains TIL1345 (*blp*::*gbs1555 shp1555*-*luxAB aphA3*), TIL1382 (*blp*::*gbs1555* P*_shp1555_*-*luxAB aphA3*) and TIL1380 (*blp*::*shp1555*-*luxAB aphA3*), respectively. Similarly, pGICB004a::*shp1509* was constructed by ligating a PCR fragment amplified with oligonucleotides SMUrgg-SpeI/SMU-EcoRI and double digested with EcoRI and SpeI, into pGICB004a. The SMU.1509 gene contains a *Eco*RI restriction site, so pGICB004a::SMU.1509 *shp1509* and pGICB004a::SMU.1509 P*_shp1509_* were constructed in two steps. First, the downstream part of SMU.1509 was amplified with oligonucleotides SMU-SpeI/SMU-2, double digested with *Eco*RI and *Spe*I, and ligated between the same restriction sites of pGICB004a. Second, the two fragments containing the fusions to the *shp* promoter were amplified with oligonucleotides SMU-1/SMU-EcoRI and SMU-1/SMUshp-EcoRI, digested with *Eco*RI and ligated into the same restriction site of pGICB004a already containing the downstream part of SMU.1509. Linearized pGICB004a::*shp1509*, pGICB004a::SMU.1509 *shp1509* and pGICB004a::SMU.1509 P*_shp1509_* were then used to transform competent cells of strain LMD-9 leading to strains TIL1386 (*blp*::*shp1509*-*luxAB aphA3*), TIL1383 (*blp*::SMU.1509 *shp1509*-*luxAB aphA3*) and TIL1384 (*blp*::SMU.1509 P*_shp1509_*-*luxAB aphA3*), respectively. Finally, TIL1042 (*amiCDE*::*erm blp*::P*_shp1299_-luxAB aphA3*), TIL1381 (*amiCDE::erm blp*::*gbs1555*::*shp1555*-*luxAB aphA3*) and TIL1385 (*amiCDE::erm blp*::SMU.1509::*shp1509*-*luxAB aphA3*) were constructed by transforming competent cells of strains TIL1038, TIL1345 and TIL1383, respectively, with chromosomal DNA from strain TIL1389 (*amiCDE::erm*).

### LC-MS/MS

*S. thermophilus* strain LMD-9 carrying the *shp1299* gene, *S. agalactiae* strain NEM316 carrying the *shp1555* gene, *S. mutans* strain UA159 carrying the *shp1509* gene, *S. thermophilus* strains TIL1345 and TIL1383 carrying the *shp* genes of *S. agalactiae* and *S. mutans*, respectively, were grown in CDM, and the culture supernatants were recovered at the end of the exponential phase. Aliquots of 5 µl of supernatant were loaded on a Pepmap C18 column (length 150 mm, 75 µm ID, 100 Å; Dionex, Voisins-le-Bretonneux) and analyzed on-line by mass spectrometry on a LTQ-Orbitrap Discovery apparatus (Thermo Fischer, San Jose). The masses of the separated molecules were first analyzed with the high resolution accuracy (10 ppm) of the Orbitrap mass analyser. Then, selected ions were fragmented in the trap by collision induced dissociation (CID) and the ion daughters were analyzed at low accuracy (250 ppm) in the linear ion trap (LTQ).

We manually extracted the ion current signals (XIC) of the masses of all monocharged peptides corresponding to the C-terminal fragments of the SHP precursors ranging from LIIVGG to FTLIMDILIIVGG for SHP1555, from IIIGGG to IVVLETIIIIGGG for SHP1509 and from FPPFG to VVIDIIIFPPFG for SHP1299. Using the sequences of the three streptococcal genomes, we checked that these peptides could not be encoded by genes other than the *shp* genes. We also checked that the XIC detected fulfilled two different criteria: (i) the retention time of the XIC detected was compatible with the hydrophobicity (GRAVY index) of the corresponding peptide and (ii), the XIC signal was absent from the supernatant of a strain that did not encode the SHP. Then, selected ions were fragmented. This approach was not successful for *S. thermophilus* expressing the *shp1509* gene of *S. mutans* (TIL1383), so a more targeted and sensitive approach was used. We searched specifically for the peptide ETIIIIGGG which has a predicted mass of 872.51 Da. First, we fragmented all the ions with a mass of 872.51+/−2 Da during the chromatographic separation and analyzed the fragments in the LTQ. Second, we extracted the MS2 XIC with a mass of 740, corresponding to the fragment b7. This transition was chosen on the basis of previous fragmentation data obtained with SHP1358 of the *S. thermophilus* strain LMD-9 [21]. The patterns of fragmentation were analyzed to identify one with all ion daughters that fitted well with the sequence of the peptide sought.

To estimate the concentration of mature SHP1358 (EGIIVIVVG) in the supernatant of *S. thermophilus* strain LMD-9, we used the corresponding heavy form of the mature peptide [NH2-EGII[V_C^13^N^15^]IVVG-OH] (Thermo, Scientific) dissolved in 5% CH_3_CN and 0.1% trifluoracetic acid, as an internal standard. The heavy peptide was added to *S. thermophilus* LMD-9 supernatant at a final concentration of 10 ng ml^–1^. The area obtained with the heavy peptide was measured and used to calculate the amount of the natural peptide.

### Luciferase Assay

Cells were grown overnight at 42°C in CDM. Cultures of 50 ml of CDM were then inoculated at an OD_600_ of 0.05 and incubated at the appropriate temperature, *i.e.* 30, 37 or 42°C. Aliquots of 1 ml were sampled at regular intervals until the culture reached the stationary phase and analyzed as described previously [21]. Synthetic peptides (Table 3), stored in lyophilized form and prepared in 5% formic acid, were added as appropriate to a final concentration of 1 µM at the beginning of the cultures. Purities of crude preparations were above 90%. Results are reported in Relative Luminescence Units divided by the OD_600_ (RLU/OD_600_). *S. thermophilus* strains TIL1345, TIL1383, TIL1038 and TIL1165 were used in cultures at 30, 37 and 42°C to assess which of these was the optimal temperature for the expression of *shp1555*, *shp1509*, *shp1299* and *shp1358,* respectively. It appeared to be 30°C for *shp1555* and *shp1509* and 42°C for *shp1299* and *shp1358* (data not shown).

**Table 3:** The *shp/rgg* loci used in this study.

	Species	Strain	Locus name^b^	Rgg name	SHP name^c^	SHP sequence^d^
Group I^a^	*Streptococcus agalactiae*	NEM316	*shp/gbs1555*	Rgg1555	SHP1555	MKKINKALLFTLIMDILIIVGG
Group II^a^	*Streptococcus thermophilus*	LMD-9	*shp/ster_1358*	Rgg1358	SHP1358	MKKQILLTLLLVVFEGIIVIVVG
	*Streptococcus mutans*	UA159	*shp/*SMU.1509	Rgg1509	SHP1509	MRNKIFMTLIVVLETIIIIGGG
Group III^a^	*Streptococcus thermophilus*	LMD-9	*shp/ster_1299*	Rgg1299	SHP1299	MKKVIAIFLFIQTVVVIDIIIFPPFG

a Group number of the SHP-associated Rgg according to the classification described in Fleuchot *et al.* [21].

b The *shp* gene is followed by the Genbank id of the *rgg* genes.

c The *shp* genes are not annotated in Genbank but were identified using BactgeneSHOW [20], except for the *shp* gene associated with *ster_1299*, which is annotated *ster_1298* in the genome of *S. thermophilus* strain LMD-9. Consequently, all the *shp* gene products are indicated with the term “SHP” followed by the number of the cognate *rgg* gene in Genbank. To unify the nomenclature, the *ster_1298* gene product was renamed SHP1299.

d The sequences of the synthetic peptides used in this study are underlined.

## Results

### Selection of Relevant *shp/rgg* Loci for the Study of Cross-talk in Streptococci

To study cross-talk among streptococci via SHP signaling peptides, we chose four *shp/rgg* loci found in the three SHP-associated Rgg phylogenetic groups [21] (Table 3). For group I, we chose the locus *shp/gbs*1555 (*rgg1555*) of *Streptococcus agalactiae* strain NEM316, present in all sequenced strains of *S. agalactiae*. The role of Gbs1555, also called RovS, in virulence has been studied but without taking into account the existence of its cognate SHP [33]. Moreover, the amino acid sequence of the predicted mature SHP of *S. agalactiae* is identical to those of the SHPs of *Streptococcus dysgalactiae* subsp. *equisimilis* and of SHP2 of *S. pyogenes* (Table S2). For group II, we chose the locus *shp/ster_1358* (*rgg1358*) of *S. thermophilus* strain LMD-9, already studied in detail [19], [21], and the locus *shp/*SMU.1509 (*rgg1509*) of *Streptococcus mutans* strain UA159 present in all sequenced strains of *S. mutans*. For group III, we chose the locus *shp/ster_*1299 (*rgg1299*) of *S. thermophilus* strain LMD-9 also found in strain JIM8232, in *Streptococcus oralis* strain SK60 and *Streptococcus tigurinus* strain 1368. These loci thus correspond to three SHP/Rgg mechanisms that have not previously been studied, including two in pathogenic streptococci of two different streptococci groups: mutans (*S. mutans*) and pyogenic (*S. agalactiae*). These pathogenic streptococci are found in the same niche in the oral cavity [28] and are therefore likely to encounter each other, and also, at least briefly, *S. thermophilus*, a species of the salivarius group that is one of the two starters used to produce yogurt. First, we studied the QS mechanisms of the loci that had not previously been studied.

### SHP/Rgg Mechanisms in Different Species of Streptococci Function Similarly

**Figure 1:**
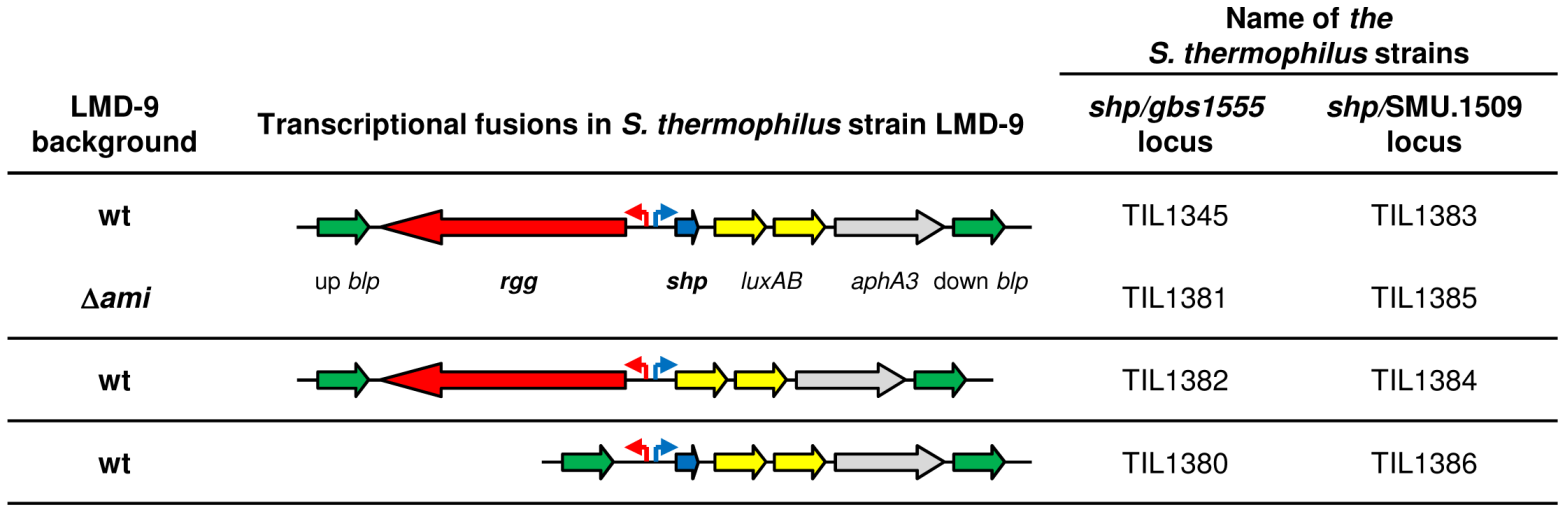
Description of strains containing P*_shp_*-*luxAB* transcriptional fusions in various genetic backgrounds. These strains were constructed in *S. thermophilus* strain LMD-9 and used to study the expression of the *shp* genes of *S. agalactiae* strain NEM316 (*shp/gbs1555* locus) and *S. mutans* strain UA159 (*shp/*SMU.1509 locus) in the presence and absence of the corresponding *shp* and *rgg* genes and in the presence and absence of the *ami* genes of *S. thermophilus* strain LMD-9.

**Figure 2:**
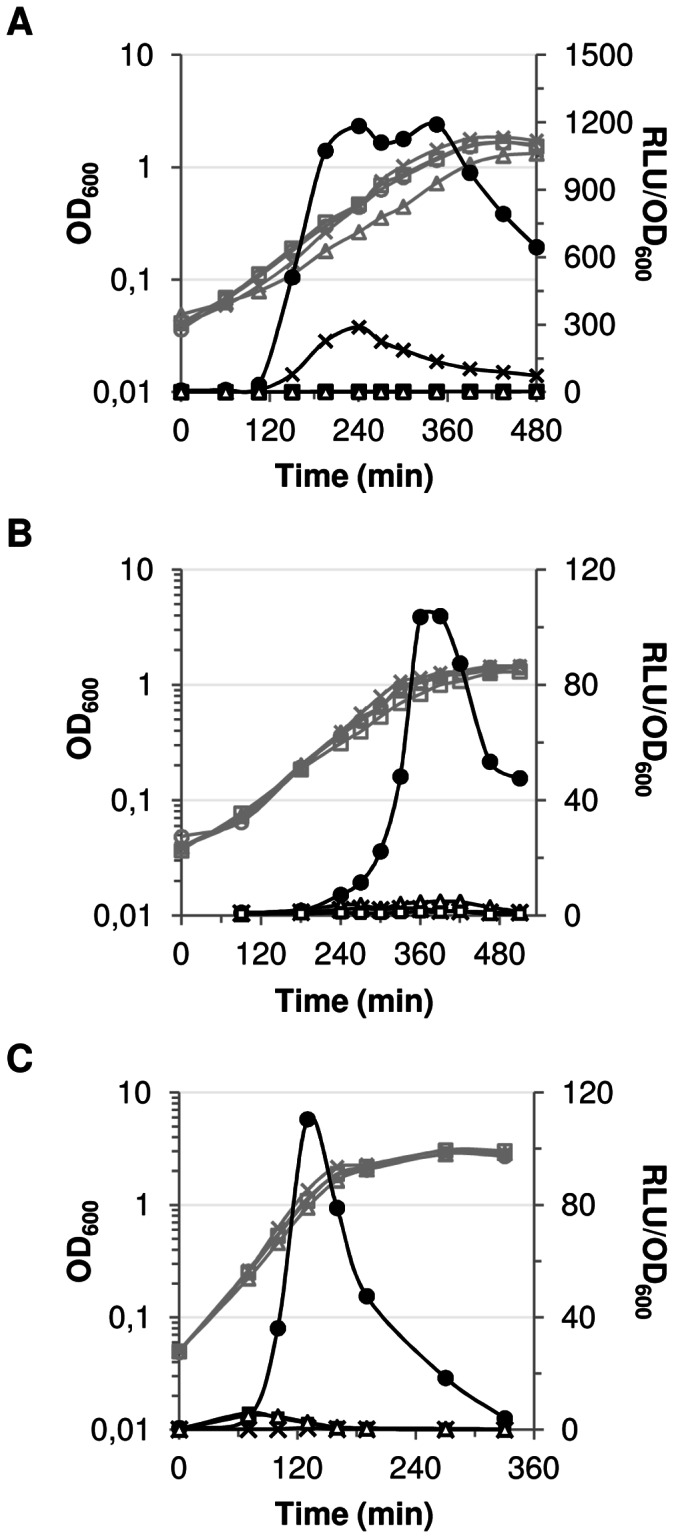
Growth and luciferase activities of strains containing P*_shp_*-*luxAB* fusions in various genetic backgrounds. Growth curves (OD_600_) are presented in gray and relative luciferase activities (RLU/OD_600_) in black. Growth and relative luciferase activities of derivatives of *S. thermophilus* strain LMD-9 grown in CDM and containing P*_shp_-luxAB* fusions of the loci *shp/gbs1555* of *S. agalactiae* (A), *shp/*SMU.1509 of *S. mutans* (B) and *shp/ster_1299* of *S. thermophilus* strain LMD-9 (C). The genetic backgrounds are indicated as follows: (•) the *shp* and *rgg* genes of the locus tested and the *ami* gene *of S. thermophilus* are present (▴) the cognate *shp* gene of the locus studied is not present, (▪) the cognate *rgg* gene of the locus studied is not present and, (**×**) the *ami* genes of *S. thermophilus* are not present. Experiments were done at 30°C for the *shp/gbs1555* and the *shp/*SMU.1509 loci and at 42°C for the *shp/ster_1299* locus. Data shown are representative of three independent experiments.

Analysis of the *shp/ster_1358* locus of *S. thermophilus* showed that the SHP1358 peptide, the Rgg1358 transcriptional regulator and the Ami oligopeptide transporter are essential for a QS mechanism that positively controls the transcription of the *shp1358* gene, creating a positive feedback loop [21]. We tested whether the auto-induction of *shp* gene expression was conserved in the SHP/Gbs1555 system of *S. agalactiae* strain NEM316, the SHP/SMU.1509 system of *S. mutans* strain UA159 and the SHP/Ster_1299 system of *S. thermophilus* strain LMD-9 (Table 3). We evaluated the activity of the *shp* promoter of each locus in *S. thermophilus* strain LMD-9, in the presence and absence of the genes encoding the three partners, SHP, Rgg and Ami. Thus, for locus *shp/ster_1299* of *S. thermophilus*, a P*_shp1299_-luxAB* fusion was introduced into the wild-type strain LMD-9 and the Δ*rgg1299*, Δ*shp1299* and Δ*amiCDE* isogenic mutants. For *shp/gbs1555* of *S. agalactiae* and *shp/*SMU.1509 of *S. mutans,* the promoter of the *shp* gene and the *shp* genes were fused, independently, to the *luxAB* genes with or without the cognate *rgg* gene and then introduced into *S. thermophilus* strain LMD-9 or its isogenic Δ*amiCDE* mutant (Figure 1). For all three loci, luciferase activity was detected when *rgg*, *shp* and *amiCDE* genes were all present (TIL1345, TIL1383, TIL1038, Figure 2A–C). If one of the genes was absent, the expression of the three P*_shp_* promoters was undetectable (Figure 2A–C) except for the *shp/gbs1555* locus of *S. agalactiae* studied in the *Δami* genetic background (TIL1381, Figure 2A): the relative luciferase activity in this case was one quarter of that in the wild-type genetic background (TIL1345). These experiments clearly demonstrated that the SHP pheromone, the Rgg regulatory protein of each locus and the Ami transporter of strain LMD-9 are required for strong expression of the three *shp* genes in the condition tested.

The pattern of activity of the different *shp* promoters differed: expression of luciferase activity started either in the middle (*shp/gbs1555* and *shp/ster_1299,* Figure 2A and C, respectively) or at the end (*shp/*SMU.1509 - Figure 2B) of the exponential growth phase. Once the maximal activity was reached, it was maintained until the end of the exponential growth phase for P*_shp1555_* (Figure 2A) but was transient for the two other promoters studied, P*_shp1509_* and P*_shp1299_* (Figure 2B and C).

These analyses indicate that the SHP/Rgg systems of both pathogenic and non-pathogenic streptococci function in a similar way and appear to be temperature (data not shown, see Materials and Methods) and growth phase-dependent in *S. thermopilus* strain LMD-9.

### The Mature Forms of SHP are Released by Cleavage of the C-terminal Part of their Precursor in Front of a Conserved Acid Residue

**Figure 3:**
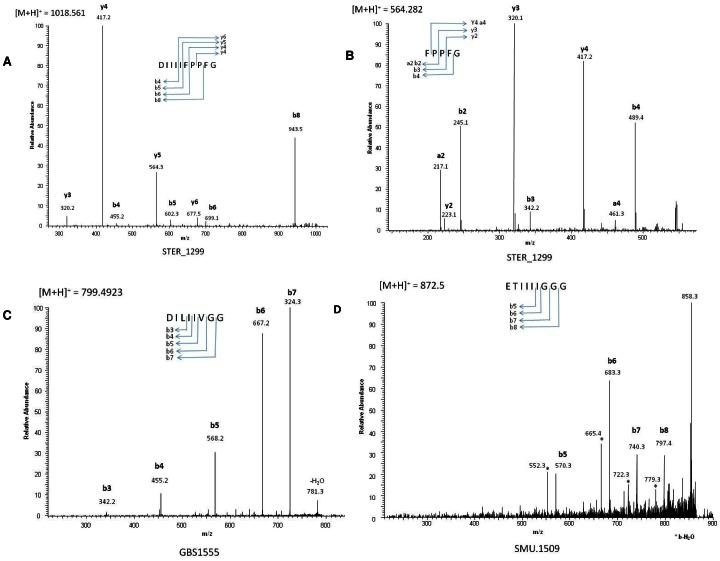
Fragmentation spectra of the ions of mature forms of SHP1299, SHP1555 and SHP1509. Fragmentation of the ions m/z 1018.56 (A) and m/z 564.28 (B) identified in the supernatant of cultures of *S. thermophilus* strain LMD-9. Fragmentation of the ions m/z 799.49 (C) identified in the supernatant of cultures of *S. agalactiae* strain NEM316 and m/z 872.5 (D) identified in the supernatant of cultures of *S*. *mutans* strain UA159. All ions were analyzed in the linear ion trap.

The mature form of the SHP1299 of *S. thermophilus* was sought directly in the supernatant of the wild-type strain LMD-9. Without purification or concentration, direct analysis of the supernantant of *S. thermophilus* LMD-9 by LC-MS/MS identified two masses corresponding to the fragments, DIIIFPPFG and FPPFG, of the SHP1299 precursor. These sequences were confirmed by fragmentation (Figure 3A–B). We used mass spectrometry to identify the sequences of the mature forms of the SHP1555 of *S. agalactiae* and SHP1509 of *S. mutans* in the supernatants of the *S. thermophilus* strains TIL1345 and TIL1383. A mass corresponding to the octapeptide DILIIVGG was identified as the mature form of the *S. agalactiae* SHP1555 produced by *S. thermophilus*. The sequence of this peptide was also confirmed by fragmentation. No mass corresponding to fragments of the precursor SHP1509 of *S. mutans* was found in supernantant of *S. thermophilus* strain TIL1383. A similar method was used to identify mature SHP peptides in the supernatants of the wild-type strains *S. agalactiae* NEM316 and *S. mutans* UA159. The production of the octapeptide DILIIVGG was confirmed for *S. agalactiae* (Figure 3C) but once again, we did not find any mass corresponding to fragments of the SHP1509 of *S. mutans.* Therefore, we predicted by analogy, that the mature SHP peptide produced by *S. mutans* was ETIIIIGGG and we used a more sensitive mass spectrometry approach (based on MS2) to detect this peptide in the supernatant of *S. mutans* UA159. This approach successfully detected one mass corresponding to this peptide (Figure 3D).

**Figure 4:**
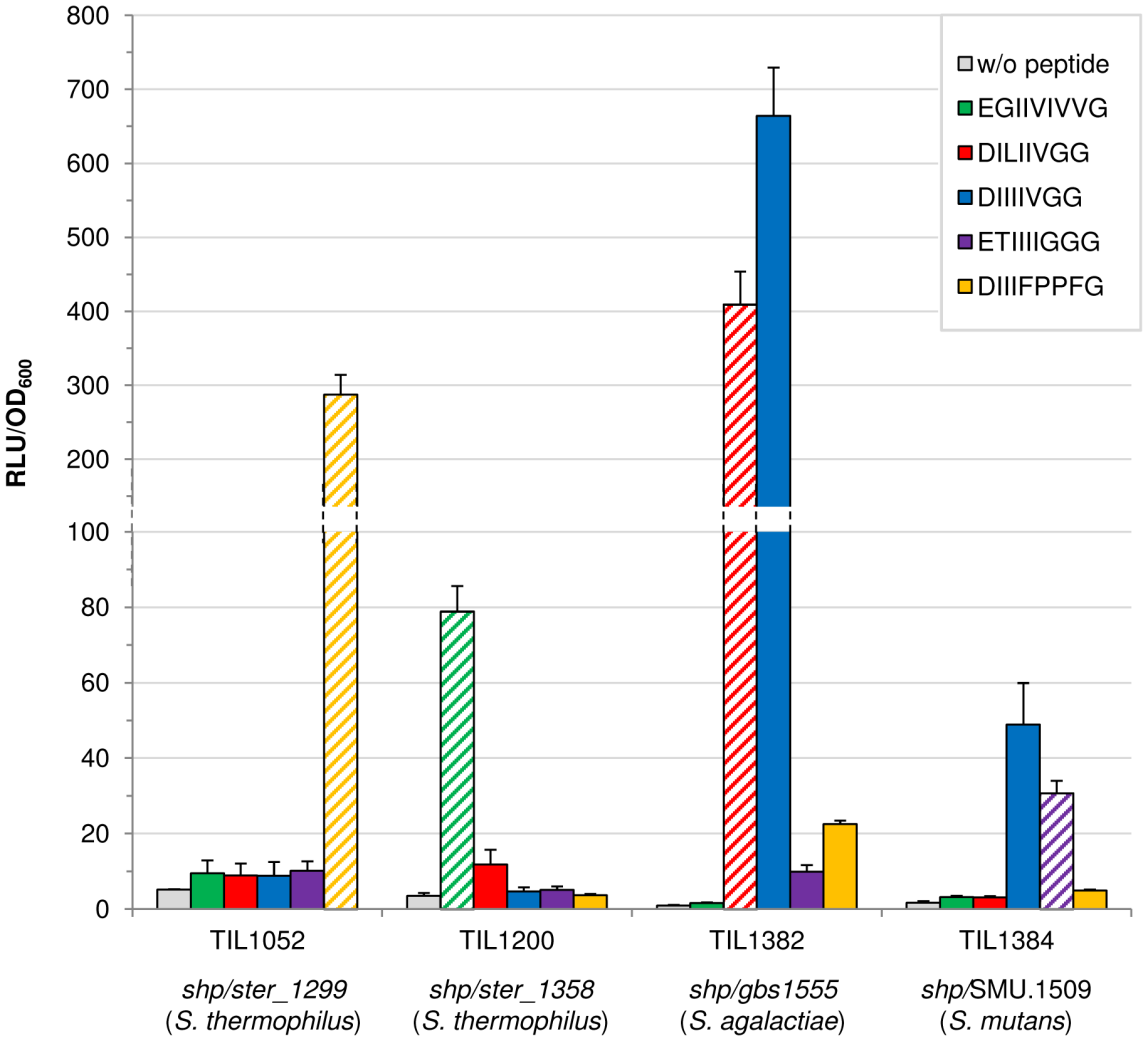
Cross-complementation of the *shp/rgg* loci with synthetic SHP pheromones. Maximum relative luciferase activities of the reporter strains TIL1052 (*shp1299*::*erm blp*::P*_shp1299_-luxAB aphA3*), TIL1200 (Δ*shp1358 blp*::P*_shp1358_-luxAB*), TIL1382 (*blp*::*gbs1555*::P*_shp1555_*-*luxAB aphA3*) and TIL1384 (*blp*::SMU.1509::P*_shp1509_*-*luxAB aphA3)* grown in the absence (grey) or in the presence of synthetic SHP peptides added at the beginning of the culture to a concentration of 1 µM: EGIIVIVVG (green), DILIIVGG (red), DIIIIVGG (blue), ETIIIIGGG (purple), DIIIFPPFG (yellow). The legitimate SHP synthetic peptide associated to the locus studied is hatched in each case.

To check that the longest peptides identified by mass spectrometry for the three loci were active, synthetic peptides with these three sequences were produced. These synthetic peptides were added to cultures of reporter strains containing a P*_shp_*-*luxAB* fusion of the corresponding locus but not encoding the cognate SHP. In all cases, the synthetic peptide functionally complemented the reporter strain (Figure 4 - TIL1052, TIL1382 and TIL1384 hatched bars).

We used mass spectrometry to evaluate the amount of SHP1358 naturally present in cultures of *S. thermophilus* LMD-9 at the end of the exponential phase of growth, which is when its gene is maximally expressed. The heavy form of SHP1358 [NH2-EGII[V_C^13^N^15^]IVVG-OH] (Thermo, Scientific) was used as an internal standard. The concentration of SHP present in the culture supernatant was estimated to be 7+/−3 ng ml^–1^.

### The SHP Pheromones Allow Cross-talk between Streptococci

SHP/Rgg QS mechanisms are widespread among species of streptococci and the amino acid sequences of the various Rgg and SHP proteins are similar (Table S2). We therefore investigated the existence of cross-talk phenomena. We used functional complementation experiments to determine whether *shp/rgg* loci can be regulated by mature SHPs with an amino acid sequence different from that of its cognate mature SHP. The four selected loci (Table 3) were studied in four reporter strains that cannot produce the cognate SHP pheromone: TIL1052 (*shp1299*::*erm blp*::P*_shp1299_-luxAB aphA3*), TIL1200 (Δ*shp1358 blp*::P*_shp1358_-luxAB*), TIL1382 (*blp*::*gbs1555*::P*_shp1555_*-*luxAB aphA3*) and TIL1384 (*blp*::SMU.1509::P*_shp1509_*-*luxAB aphA3)*. Five synthetic peptides were used: four corresponding to these *shp/rgg* loci (Table 3) and one with the sequence DIIIIVGG, which is identical to the SHP of *S. pyogenes,* called SHP3, and differs from one amino acid to that of *S. agalactiae* (Table S2). These five peptides were added independently to cultures of the four reporter strains. For the two loci of *S. thermophilus* (TIL1052 and TIL1200), no significant induction of luciferase activity was detected with any of the non-cognate synthetic peptides. However, for the two loci from the two pathogenic streptococci (TIL1382 and TIL1384), luciferase activity was induced more strongly by the illegitimate peptide DIIIIVGG than by the cognate peptide (hatched bars); the other synthetic SHPs had no detectable effects (Figure 4).

## Discussion

SHP/Rgg cell-cell communication mechanism has been deciphered using the SHP/Rgg1358 locus of *S. thermophilus* from group II [21] and the SHP2/Rgg2 and SHP3/Rgg3 of *S. pyogenes* from group I as models [23], [24]. We have increased the number of examples of this mechanism by studying another locus in *S. thermophilus* (group III), one of *S. agalactiae* (group I) and one of *S. mutans* (group II). We confirmed that the *shp* genes are targets of the mechanism that relies on Rgg, SHP and Ami. These validations were performed in *S. thermophilus i.e.* in a heterologous background for the *shp/rgg* loci of *S. agalactiae* and *S. mutans*. Thus, *S. thermophilus* is able to secrete, process and import SHPs from other species efficiently. Except for the *shp1555* gene of *S. agalactiae* in the *ami*-deleted mutant, no expression of the three *shp* genes was observed in the *shp–*, *rgg–* or *ami*-deleted mutants although *S. thermophilus* strain LMD-9 contains six *shp*/*rgg* loci, including at least two that are active in our conditions. This implies that the interactions between SHP and Rgg and between Rgg and its DNA target are highly specific. The expression of the *shp1555* gene in the *ami*-deleted mutant was only one quarter of that in the wild-type genetic background; possibly, the precursor SHP is able to activate the Rgg regulator and bypass the *ami* deletion or the SHP precursor is processed intracellularly and able to activate the Rgg regulator. The SHP/Rgg mechanism for the *S. mutans* and *S. agalactiae* systems need to be confirmed in their homologous backgrounds. Appropriate experiments are in progress with the *shp/gbs1555* locus of *S. agalactiae* (D. Perez-Pascual, unpublished results). It is extremely likely that the proposed mechanism involving the *shp*, *rgg* and *ami* genes in these pathogenic bacteria will be confirmed, but it would be interesting to document the kinetics of expression of *shp*. In *S. thermophilus*, expression of the *shp* gene of *S. agalactiae* started early during the exponential phase of growth whereas that of the *shp* gene of *S. mutans* started at the beginning of the stationary phase. If confirmed in the homologous background, it will suggest that there are additional components contributing to the control the expression of the *shp* genes. In conclusion, this validation of the cell-cell communication mechanism for three new *shp*/*rgg* loci, including one from a group that had not previously been studied (group III), suggests that the main components (SHP, Rgg and Ami) are conserved in all SHP/Rgg mechanisms.

The mature forms of SHP1299, SHP1555 and SHP1509 identified directly in *S. thermophilus*, *S. agalactiae* and *S. mutans* supernatants were each the C-terminal part of the precursor and start with an acid amino acid (Asp or Glu). These amino acids are conserved in nearly all SHP identified from streptococci genome sequences; the exceptions are one in *S. thermophilus* strain LMG18311 (Rgg Stu0182-associated SHP) and another in strain CNRZ1066 (Rgg Str0182-associated SHP) that contain a Cys residue at this position [21]. Naturally secreted SHP1358 also starts with a Glu [21]. The activity of SHP2 in *S. pyogenes* is maintained if the Asp amino acid at this position is substituted with a Glu, but not with an amide-bearing residue [23]. Therefore, all mature SHPs are expected to have an Asp or Glu at their N terminus. These conserved residues seem to be required for the recognition of the precursor by the protease involved in their maturation and the activity of the mature SHP. The Eep membrane protease is involved in the production of mature SHP by *S. thermophilus* [21] and *S. pyogenes* [23]. It has not been established whether or not this role is direct. Nevertheless, Eep-encoding genes are present in all streptococci genomes, and map in a conserved environment, so it is highly probable that this role is common to all streptococci. The amino acid sequence of the SHP1555 of *S. agalactiae* produced by *S. thermophilus* and by *S. agalactiae* were identical, consistent with the conservation of the role of Eep, and the maturation more generally. In *Enterococcus faecalis*, the sex pheromones are matured by Eep [34], [35], [36], but there is no conservation of such acid amino acids. The maturation of the XIP, another family of signaling peptides produced by streptococci and that are involved in the triggering of competence, seems less well conserved. Indeed, Eep is involved in the production of the XIP of *S. thermophilus* but not in that of *S. mutans*. The sequences of the XIP peptides are less conserved than those of the SHP peptide, and this may explain the involvement of different proteases in their maturation.

We detected a shorter mature form of SHP1299 in the supernatant of *S. thermophilus*. Shorter forms were not detected in other supernatants but it does not indicate that there are not present in small amounts. This probably means that these linear non-modified peptides are subject to degradation by, at least, aminopeptidases present in the extracellular medium. We have already observed such N-terminal degradation with ComS [37] indicating the existence of a significant aminopeptidase activity at the surface of streptococci.

The cross-talk experiments with four *shp*/*rgg* loci and five synthetic SHPs showed a generally high specificity of the SHP/Rgg interaction. Only one peptide, SHP3 from *S. pyogenes*, was able to induce the expression of the *shp* genes from the other species. This result for *S. agalactiae* was not surprising because the two peptides differ only at the third residue, and the difference is very minor (DIIIIVGG/DILIIVGG). *S. pyogenes* encodes both peptides, and they can stimulate the expression of their targets to similar levels [23]. The cross-talk result with *S. mutans* was more surprising: the amino acid sequences of the SHPs are more divergent and they do not belong to the same group despite both containing a hydrophobic stretch of isoleucines (DIIIIVGG/ETIIIIGGG). The SHP of *S. agalactiae* was not able to cross talk with the *S. mutans* system and *vice versa* indicating that the presence of the four isoleucine residues are critical only for the *S. mutans* system, and that the specificity of the interaction is complex. Mature SHP3 peptide can be produced by three different species of streptococci *i.e. pyogenes*, *pneumoniae* and *thermophilus*, and the mature SHP peptide of *S. agalactiae* can be produced by two other species of streptococci, *pyogenes* (SHP2) and *S. dysgalactiae* (Table S2). This suggests that if present in the same environment, these streptococci can potentially interact with each other through their SHP/Rgg systems. It would be interesting to investigate this possibility with co-cultures in an ecosystem model. Such interactions may be of great significance to the co-operation or competition between streptococci species.

## Supporting Information

Table S1 **Primers used in this study.**
(DOCX)

Table S2 **Comparison of the sequences of six similar SHP pheromones.**
(DOCX)
