# Supplementary material for: Rgg-Associated SHP Signaling Peptides Mediate Cross-Talk in Streptococci
Source: PLoS One. 2013 Jun 11;8(6):e66042. doi: 10.1371/journal.pone.0066042 (PMC3679016; doi:10.1371/journal.pone.0066042)
Supplement: Table S1 — Primers used in this study. (DOCX) [file pone.0066042.s001.docx]

**Table S1. Primers used in this study.**

| **Primer** | **Sequence (5’ 🡪 3’)^a,b^** |
| --- | --- |
|  |  |
| AphA3-F | ccagcgaaccatttgag |
| AphA3-R | gttgcggatgtacttcag |
| Erm-F | GGGACCTCTTTAGCTCCTTGG |
| Erm-R | GGAGATAAGACGGTTCGTGTTCG |
| amiCDE_up-F | CGGCTTTGACCTACACG |
| amiCDE_up-R | CTActgacagcttccaaggagctaaagaggtcccAAGGCGATGGTTGGTAGTG |
| amiCDE_down-F | gcaagtcagcacgaacacgaaccgtcttatctccTGAGGAAAAAGCACCAC |
| amiCDE_down-R | AAGTGACCTGGGCGTAC |
| shp1299_up-F | AATCGTTGATAATCATCCC |
| shp1299_up-R | CTACTGACAGCTTCCAAGGAGCTAAAGAGGTCCCGCTGTATCCTCCTAATCA |
| shp1299_down-F | GCAAGTCAGCACGAACACGAACCGTCTTATCTCCTTATCTTTCCCCCTTTTGG |
| shp1299_down-R | TTGACAAATACAAAGAAAGTC |
| ster_1299-SpeI | AACTACTAGTGTATGTTTCAAGTATCG |
| ster_1299- EcoRIA | CTCTTTATAGAATTCACC |
| ster_1299- EcoRIB | GAAGAATTCCAGAGACTATCCCAATA |
| ster_1299-HindIII | AAGAAGCTTGATTTTTTCTCGAAGTGTC |
| Pshp1299-EcoRI | GAAGAATTCACTTTTTTCATGCTGTATCC |
| Pshp1299-SpeI | AACTACTAGTACATATGCCTGTTCTCC |
| GBS-EcoRI | GAAGAATTCTTATCCACCAACGATAAT |
| GBS-SpeI | AACTACTAGTGCACTCATTAGCATTCTT |
| GBSrgg-SpeI | AACTACTAGTATCTTCTTAGTGTTTTTCCT |
| GBSshp-EcoRI | GAAGAATTCATTTTTTTCATGACTATCTCCT |
| SMU-1 | GCATCAATCAGTTTATCTC |
| SMU-2 | GAGACGGTCAGATTTTT |
| SMU-EcoRI | GAAGAATTCCTACCCACCGCCGATA |
| SMU-SpeI | AACTACTAGTAAGAGAGATTTTTATCCC |
| SMUshp-EcoRI | GAAGAATTCTTATTTCTCATGTTTTTCTC |
| SMUrgg-SpeI | AACTACTAGTCCTTCATTTTTAGACCGC |
|  |  |

1. Restriction enzyme recognition sequences are underlined.
2. Sequences complementary to the erythromycin cassette are underlined with dashes.
