# Supplementary material for: Rgg-Associated SHP Signaling Peptides Mediate Cross-Talk in Streptococci
Source: PLoS One. 2013 Jun 11;8(6):e66042. doi: 10.1371/journal.pone.0066042 (PMC3679016; doi:10.1371/journal.pone.0066042)
Supplement: Table S2 — Comparison of the sequences of six similar SHP pheromones. (DOCX) [file pone.0066042.s002.docx]

**Table S2. Comparison of the sequences of six similar SHP pheromones.**

| **Species and strain** | **GeneBank name**  **of the cognate *rgg* gene^a^** | **SHP sequence**^b,c^ |
| --- | --- | --- |
|  |  |  |
| *Streptococcus agalactiae* NEM316 | *gbs1555* (SHP1555) | MKKINK-ALLFTLIMDILIIVGG |
|  |  |  |
|  |  |  |
| *Streptococcus dysgalactiae* GGS_124 | *sdeg_0529* | MKKINK-ALLLTLIMDILIIVGG |
| *Streptococcus pyogenes* NZ131 | *spy49_0415* (SHP2) | MKKVNK-ALLFTLIMDILIIVGG |
|  |  |  |
|  |  |  |
| *Streptococcus pyogenes* NZ131 | *spy49_0449c* (SHP3) | MKKISKFLPILILAMDIIIIVGG |
| *Streptococcus thermophilus* LMG18311 | *stu1044* | MEKVSKILPILILVMDIIIIVGG |
| *Streptococcus pneumonia* R6 | *spr0960* | MKKISKFLPILILAMDIIIIVGG |
|  |  |  |

1. The name of the cognate SHP is in brackets.
2. The amino acids which differ from the sequence of SHP1555 are underlined.
3. The putative mature forms of the SHP are framed.
